# Comparative proteomic analysis provides insights into wood formation in immature xylem at different ages in *Eucalyptus urophylla* × *Eucalyptus grandis*

**DOI:** 10.3389/fpls.2024.1431164

**Published:** 2024-10-30

**Authors:** Guo Liu, Guowu Zhang, Zhihua Wu, Wanhong Lu, Yan Lin, Chubiao Wang, Xiuhua Shang, Anying Huang, Jianzhong Luo

**Affiliations:** ^1^ Research Institute of Fast-Growing Trees, Chinese Academy of Forestry, Zhanjiang, China; ^2^ State Key Laboratory of Tree Genetics and Breeding, Chinese Academy of Forestry, Beijing, China; ^3^ College of Coastal Agriculture Sciences, Guangdong Ocean University, Zhanjiang, China

**Keywords:** proteomics, immature xylem, wood formation, gene expression, *Eucalyptus urophylla × Eucalyptus grandis*

## Abstract

**Introduction:**

Wood formation is a crucial developmental stage in the life cycle of a woody plant; this process has substantial scientific research implications and practical applications. However, the mechanisms underlying woody plant development, especially the process of wood formation, remain poorly understood. As eucalyptus is one of the fastest growing tree species in the world, understanding the mechanism of wood formation in eucalyptus will greatly promote the development of molecular breeding technology for forest trees.

**Results:**

In this study, we investigated the proteomic profile of immature xylem at four different ages of *Eucalyptus urophylla* × *Eucalyptus grandis* (*E. urograndis*) using iTARQ technology. We identified 5236 proteins and 492 differentially abundant proteins (DAPs). The expression profiles of the DAPs corresponding to coding genes associated with wood formation were assessed using qRT-PCR. From the different expression profiles, it is inferred that the genes encoding kinesin, CDKD3, EXPA13, EXPA2, XTH27, EGases, UGT76E2, LAC, CCoAMT, CesA3, PAL, and CAD may undergo posttranscriptional regulation (PTR). Additionally, the genes encoding EIN2, ETR, MC4-like, and XCP may undergo posttranslational modifications (PTMs).

**Conclusions:**

We investigated changes in wood formation-related proteins at the protein abundance level in the immature xylem of *E. urograndis*, thereby elucidating potential regulatory mechanisms of key proteins involved in eucalyptus wood formation. This study may provide theoretical guidance for further research on molecular breeding techniques and genetic improvement related to the cultivation of rapidly growing and high-quality trees.

## 1 Introduction

Wood is the secondary xylem of forest trees and serves as the primary structural component for energy storage in perennial woody plants. Additionally, it plays a crucial role as an essential industrial raw material and renewable bioenergy source. Wood not only finds application in various human-made products but also holds significant importance in national economies and ecological development. The export of timber has been severely restricted by major timber-producing countries due to international environmental protection pressures. Consequently, China, which is the second-largest consumer and largest importer of timber globally, faces considerable challenges related to timber resource scarcity and safety issues (Zhang, 2022). To address these concerns and safeguard the timber industry’s economy, there is a pressing need to establish rapidly growing high-quality plantations while increasing forest resources and timber supplies. However, conventional theories and breeding techniques encounter technical limitations when attempting to increase wood growth rates and properties because of the long growth cycles and high heterozygosity of trees. Progress in wood-breeding techniques aimed at improving wood properties has been relatively slow. Genetic engineering technology combined with modern molecular biology methods offers effective means for enhancing wood quality within forests by improving specificity and feasibility and expediting genetic enhancements (Li, 2019). Molecular biology techniques aim to leverage the molecular mechanisms involved in wood formation processes to significantly increase wood quality through the study of key gene regulatory mechanisms that govern this process. The process of wood formation is a highly intricate and dynamic phenomenon that is tightly regulated by gene expression. It is imperative to determine the pivotal regulatory factors and mechanisms involved in wood formation for the effective utilization of molecular biology techniques aimed at enhancing wood properties, accelerating the wood growth rate, and maximizing wood yield. Wood formation encompasses various biological processes, including cell division and differentiation, cell expansion, the deposition of the secondary cell wall (SCW), and programmed cell death (PCD) (Luo and Li, 2022; Ye and Zhong, 2015).

The development of cells during wood formation is strongly influenced by cell division and differentiation in the plant meristem (Luo and Li, 2022). Dedifferentiation, trans differentiation, and redifferentiation occur in xylem mother cells and developing xylem tissues within the immature xylem to complete the reconstruction of the secondary vascular system after girdling (Zhang et al., 2023). Extensive research has been conducted on the regulation of cell division and differentiation in plant meristem cells in the fields of forest biology and wood formation. The interaction between CLAVATA (CLV) and WUSCHEL (WUS) forms a feedback regulatory mechanism that significantly influences stem cells in the shoot apex meristem (Su et al., 2020). Another feedback regulatory mechanism involving WUSCHEL-related homeobox 5 (WOX5) and CLAVATA3/ESR-related 40 (CLE40), which play crucial roles in maintaining stem cell activity in the apical meristem, has been discovered (Zhang et al., 2019). The CLE41/44-TDR/PXY-WOX4 pathway in the vascular cambium is responsible for maintaining cambium activity and promoting proliferation (Galibina et al., 2023). Our research team also discovered that numerous genes associated with cell division and differentiation play crucial roles in the molecular-level maintenance of activity in the vascular cambium. For example, expansin, kinesin, histone, cyclin, and other related genes are highly expressed in the vascular cambium of young eucalyptus trees. However, as tree age increases, the expression level of these genes decreases (Liu et al., 2023). Furthermore, *Populus tomentosa* and *Betula pendula* exhibit periodic changes in gene expression related to the cell division cycle (CDC), similar to the annual cycle activity of the cambium (Li et al., 2009, 2022). Zhang et al. (2021) identified twelve cell-related genes involved in cell division during xylem development. The regulation of cell division is governed by a family of serine/threonine kinases known as cyclin-dependent kinases (CDKs), which control progression through the cell cycle (Carneiro et al., 2021). *Arabidopsis thaliana* possesses more than seventy regulators involved in the cell cycle, including CDKs and cyclins (Qi and Zhang, 2019). Additionally, it remains unclear how signal transduction is mediated by most of these hormones, including auxin, cytokinin (CK), brassinosteroids (BRs), ethylene (ET), gibberellins (GAs), and abscisic acid (ABA), during the regulation of cell division and differentiation in tree trunk growth (Du et al., 2023; Luo and Li, 2022).

Cell expansion is a crucial process in cell growth and wood formation, as it ultimately determines the final shape and size of cells within wood tissues. According to Luo and Li (2022), the regulation of xylem cell expansion during wood formation involves multiple factors, such as plant hormones, expansins, hydrolases, and other molecular networks. Additionally, Wang et al. (2019) identified *KNAT7* (*KNOTTED ARABIDOPSIS THALIANA7*), a Class II KNOX-like homeobox protein in rice (*Oryza sativa*) that interacts with various partners to govern cell expansion. Understanding the molecular mechanisms underlying xylem cell expansion has significant implications for tree improvement.

The SCW is a specialized cell structure that forms through the specific differentiation of xylem cells after their radial growth ceases. Compared with the primary cell wall (PCW), the PCW has distinct characteristics and compositions. The SCW is thicker, multilayered, and contains a significantly greater cellulose content (60% in SCW versus 20–30% in PCW), with a more regular arrangement of cellulose microfibrils (Wang et al., 2021). Investigating the functions of genes and transcription factors (TFs) involved in regulating the biosynthesis pathway of major components in the SCW can provide a molecular basis for effectively enhancing wood quality and yield in targeted directions. Genes encoding enzymes involved in the cellulose, hemicellulose, and lignin biosynthesis pathways have been isolated and identified across various tree species, while extensive research has been conducted on the transcriptional regulatory mechanisms underlying the synthesis of each component. Cellulose serves as the structural framework and crucial constituent of plant cell walls; its complex synthesis process is regulated by multiple enzymes and genes, among which the cellulose synthase (CesA) enzyme plays a pivotal role (Hill et al., 2014). The biological functions of *CesA* genes in multiple tree species have been elucidated through isolation and analysis by numerous researchers. For example, *P. trichocarpa* possesses 17 *CesA* genes, and expression data have revealed the involvement of five specific *CesA* genes (*PtiCesA4*, *7A*, 7B, 8A, and 8B) in SCW biosynthesis (Xu et al., 2021). *Eucalyptus grandis* has 16 *CesA* genes, four of which (*EgCesA1*, *2*, *3*, and *6*) are associated with SCW biosynthesis (Liu et al., 2021). Additionally, *ClCesA1* and *ClCesA2* in *Cunninghamia lanceolata* may participate in SCW formation (Pang et al., 2015). *Pinus taeda* has 14 *CesA* genes, among which three (*PtCesA1*, *2*, *3*) are coexpressed at relatively high levels during SCW biosynthesis (Hou et al., 2023; Nairn and Haselkorn, 2005). The genes *BlCesA1*, *3*, and *4* in *B. luminifera* primarily participate in SCW formation (Huang et al., 2014). Among lignocellulosic biomass, hemicellulose is the second most abundant renewable natural polysaccharide after cellulose (Rao et al., 2023). It is synthesized by glycosyltransferases located in the Golgi body membrane and matrix. Researchers have isolated and identified several glycosyltransferases involved in wood polysaccharide (Nishikubo et al., 2011; Qin et al., 2011), xyloglucan (Ishida and Yokoyama, 2022; Kong et al., 2015), and mannan (Aspeborg et al., 2005) biosynthesis. These findings suggest that these glycosyltransferases may contribute to wood formation. Lignin, a major component of wood and an integral part of SCWs, directly influences wood strength and density. Phenylpropanoid metabolites are crucial secondary compounds associated with lignin biosynthesis. Researchers have identified eleven proteases involved in converting phenylalanine into three lignin monomers (H, G, and S-lignin). Numerous studies have extensively investigated lignin biosynthesis in various tree species, including poplar, eucalyptus, and pine. Kushwah et al. (2020) and Zhang et al. (2021) confirmed that XTHs play a significant role in the formation of SCWs in vascular tissues. The majority of key enzymes related to lignin biosynthesis have been successfully cloned and characterized (Souza et al., 2023; Yao et al., 2021). Through biotechnology approaches, researchers have also conducted functional studies on key enzyme-encoding genes that regulate pathways associated with cellulose, hemicellulose, lignin, and pectin synthesis to selectively modify the content of each component within wood.

After lignification, tubular molecules and fibrocytes undergo PCD, which represents the final step in the maturation of xylem cells (Li et al., 2020). PCD associated with plant development is exemplified by cell death during tracheary element (TE) differentiation (Han et al., 2012). In the process of xylem development, xylem vessels rapidly undergo a vacuole-splitting type of cell death shortly after differentiation from the vascular cambium, whereas xylem fibers experience a nonvacuole-splitting type of cell death. PCD in xylem cells is characterized by genomic DNA degradation. Studies have demonstrated that several nucleases (DNases) participate in the PCD of xylem cells. Calcium-dependent DNases (CaNs) are involved in nuclear DNA degradation and contribute to PCD in secretariat cavity cells in *Citrus* species (Bai et al., 2020). Proteases also play a regulatory role in various plant PCD processes (Buono et al., 2019). Caspase-3-like activity is involved in multiple aspects of both PCD and plant development (Sahoo et al., 2023). Additionally, phytohormones are implicated in PCD-associated processes (Jiang et al., 2021); however, the precise molecular and biochemical mechanisms underlying the role of plant hormones as signals for tissue- and organ-specific PCD remain to be elucidated.

Eucalyptus is a globally significant hardwood resource and an essential timber species for industrial purposes. Rapidly growing cultivars have been extensively planted in South China, bridging the gap between the wood supply and demand while ensuring wood safety. The growth of eucalyptus stems involves secondary growth and development, providing suitable materials for studying wood formation regulation. In this study, iTraq quantitative proteomics technology was employed to analyze proteins associated with secondary xylem differentiation in the immature xylem of *Eucalyptus urophylla* × *Eucalyptus grandis* (*E. urograndis*) at different ages in response to variations in tree age. Proteins were quantified, and gene expression levels were determined via qRT-PCR analysis to investigate the process of wood formation in *E. urograndis* at different ages corresponding to various degrees of maturity. This information serves as a theoretical reference for understanding the molecular mechanisms underlying eucalyptus wood formation, contributing to improved forest breeding standards, promoting forest development, and enhancing wood safety.

## 2 Materials and methods

### 2.1 Plant materials

Plant materials were collected from 12 clonal trees of *E. urograndis* (DH32–29) of four different ages (3, 6, 9, and 11 years), which were cultivated at the South China Experimental Nursery. Three biological replicates were obtained for each age. The study site was located at an altitude of 90 m with coordinates of 111°09′ E and 21°26′ N. Tissue samples were obtained from the immature xylem, specifically the outer cambium layer (1–2 mm thick) covering the stem, consisting of xylem mother cells and developing xylem tissue. These samples were collected on September 18, 2020, at the diameter at breast height (DBH) level of each tree. To collect the tissue samples, a section of about 15 × 10 cm^2^ bark was removed from each tree’s stem surface. Each tree provided two pieces of bark oriented toward the south and north. The exposed tissues on the stems were gently scraped using a sterile blade and immediately frozen in liquid nitrogen for subsequent analysis. Detailed information about all the samples can be found in **Supplementary Table 1**.

### 2.2 Protein extraction and quantitative detection

The 12 immature xylem of *E. urograndis* was subjected to protein extraction using the SDS-phenol method, followed by storage at –80°C. The concentration of the extracted proteins was determined via the Bradford method (Kielkopf et al., 2020), with bovine serum albumin (BSA) used as the reference standard. Enzymography was conducted to detect and quantify protein concentrations on the basis of a standard curve (Vandooren et al., 2013). Protein quality assessment was performed through SDS-PAGE.

### 2.3 Trypsin digestion and iTRAQ labeling

After 200 µg of protein was quantified, the mixture was transferred to a centrifuge tube. Subsequently, 5 µL of a 1 mol/L dithiothreitol (DTT) solution was added and thoroughly mixed, followed by incubation at 37°C for 1 h. After incubation, 20 µL of 1 mol/L IAA solution was added, and the two solutions were thoroughly mixed. The reaction was then allowed to proceed at room temperature in the dark for 1 h. Afterwards, all the samples were absorbed and transferred to an ultrafiltration tube. After the contents were centrifuged, the liquid phase was discarded. Next, 100 µL of UA (8 mol/L urea and 100 mmol/L Tris-HCl, pH 8.0) was added to the ultrafiltration tube and centrifuged again. The liquid phase was discarded, and this step was repeated twice. Then, 100 µL of a 0.5 mol/L triethylammonium bicarbonate buffer volatile buffer (TEAB) solution was added to the ultrafiltration tube. The contents were centrifuged, and the liquid phase was discarded; this process was repeated three times consecutively. Finally, trypsin was added to the ultrafiltration tube at a protein-to-enzyme ratio of 50:1 for enzymatic digestion at an incubation temperature of 37°C for about 12–16 h.

About 100 µg peptide segments from each group were collected following isobaric tags for relative and absolute quantitation (iTRAQ) Reagent-8Plex Multiplex Kit instructions before labeling them accordingly. Next, a subset of samples was mixed prior to analysis using liquid chromatography-tandem mass spectrometry (LC-MS/MS) to assess iTRAQ labeling efficiency.

### 2.4 HPLC fractionation

After the label was removed, the sample was reconstituted with 100 µL of mobile phase A (98% H_2_O, pH 10), vortexed, and centrifuged at 14,000 ×g for 20 min. The supernatant was collected, and the sediment was discarded. Blank centrifuge tubes (1.5 mL) were prepared and sequentially labeled from 1–60 to collect components separated into groups numbered accordingly. The flow rate and separation gradient were proportionally distributed. Starting from the fifth minute, the eluents were collected every 1.5 min in corresponding numbered centrifuge tubes until all the samples had been collected. The collected samples underwent vacuum refrigeration and centrifugal drying before being reconstituted with a solution containing 0.5% formic acid (FA). All sixty components were combined after drying and mixing thoroughly before undergoing further analysis via LC-MS/MS following extraction of about one hundred micrograms of peptide segment per group using the iTRAQ Reagent-8Plex Multiplex Kit (AB Sciex). Finally, several mixed samples were analyzed to assess iTRAQ labeling efficiency.

Reverse-phase separation was conducted at high pH using an HPLC liquid-phase system with a nanolift flow rate. The labeled samples were subsequently dissolved in 20 µL of 2% methanol and 0.1% formic acid, followed by centrifugation at 12,000 ×g for 10 min. After the supernatant was collected, it was subjected to analysis using the sandwich method with a sample volume of 10 µL. The loading pump flow rate was set at 350 nL·min^–1^ for 15 min, while the separation flow rate remained constant at 300 nL·min^–1^. Prior to further analysis, SDS-PAGE was performed as a quality control measure to ensure clear protein bands without degradation.

Each group received an equal amount of protein (typically about 100 µg) for enzymatic digestion. To increase the enzymatic hydrolysis efficiency, reduction, alkylation, and secondary enzymatic hydrolysis were carried out before proceeding with the main process. The resulting peptides from enzymatic hydrolysis were subjected to desalting treatment followed by an iTRAQ/TMT labeling reaction, in which heteroisotopically labeled reporter groups were added to accurately quantify peptide segments from different samples.

To simplify peptide complexity within a single run and increase overall protein identification throughput, additional component separation of mixed labeled peptides was performed using HPLC with a high-pH reverse C18 column, which resulted in about 8–20 components based on the complexity of the protein sample.

### 2.5 LC-MS/MS analysis

The column was initially equilibrated with 95% liquid A (0.1% FA/H_2_O) for preconditioning. Each sample subsequently underwent capillary high-performance liquid chromatography separation followed by analysis using a Q Exactive mass spectrometer (ThermoFisher Scientific, MA, USA) that was coupled to Easy nLC (ThermoFisher Scientific, MA, USA) for 60 min. The mass spectrometer was operated in positive ion mode. MS data were acquired using a data-dependent top 10 method that dynamically selected the most abundant precursor ions from the survey scan range of 350–1800 m/z for HCD (Higher energy collisional dissociation) fragmentation. Survey scans were acquired at a resolution of 70000 with an AGC (Automatic gain control) target of 3e^6^ and a maxIT (maximum injection time) of 45 ms. MS^2^ scans were acquired at a resolution of 17500 with an AGC target of 2e^5^ and a maxIT of 45 ms, and isolation window was 2.0 m/z.

### 2.6 Database search and bioinformatics analysis

The MS data were converted to MGF files using Proteome Discovery 1.4 software (ThermoFisher Scientific, Waltham, MA, USA) and analyzed utilizing the Mascot search engine (Matrix Science, London, UK; version 2.3.2). The Mascot database derived from the transcriptome of *E. urograndis* (SRP425632) was employed for protein identification. A fragment ion mass tolerance of 0.050 Da and a parent ion tolerance of 10.0 ppm were applied during the Mascot database search process. The FDR of protein identification and peptide-spectrum match identification was set to the 0.01 threshold level.

The annotation of the identified proteins was performed using commonly employed functional databases, such as the Clusters of Orthologous Groups of proteins (COG), Gene Ontology (GO), and Kyoto Encyclopedia of Genes and Genomes (KEGG) databases. The identification of differentially abundant proteins (DAPs) was based on a fold change in abundance greater than 1.2-fold, accompanied by a statistically significant *P* value less than 0.05 according to the Student’s *t* test results. Subsequently, functional enrichment analyses encompassing GO and KEGG pathway analyses were performed on these DAPs.

### 2.7 qRT-PCR

We selected 50 protein-coding genes for quantitative real-time polymerase chain reaction (qRT-PCR) analysis. The primer pairs were designed via Primer 6.0, and total RNA was extracted from the 11 tissue samples using an RNAprep Pure Plant Kit (TIANGEN, Beijing, China). The RNA quality of all the samples was assessed with a NanoDrop 2000 spectrophotometer (Thermo Fisher, Waltham, MA, USA). qRT-PCR analysis was performed using a QuantStudio StepOne Plus Real-Time PCR System (Thermo Fisher Scientific, Inc., USA) following the manufacturer’s instructions. Reverse transcription amplification was conducted using the SynScript^®^III RT SuperMix for qPCR Kit (Beijing Tsingke Biotech Co., Ltd., China). The resulting cDNA product obtained from reverse transcription was subsequently diluted fourfold and used as a template for qPCR amplification with ArtiCan^CEO^ SYBR qPCR Mix (Beijing Tsingke Biotech Co., Ltd., China). A total volume of 20 μL containing 1 μL of cDNA template, 10 μL of ArtiCanCEO SYBR qPCR Mix (Beijing Tsingke Biotech Co., Ltd., China) and 1 μL each of forward and reverse primers (10 μM) was used for qRT-PCR. The PCR cycling parameters consisted of an initial predenaturation step at 95°C for 5 min, followed by 40 cycles comprising denaturation at 95°C for 15 s, annealing at 60°C for 20 s, and extension at 72°C for 20 s. Subsequently, a melting phase was performed with steps including denaturation at 95°C for 15 s, annealing at 65°C for 1 min, and then gradually increasing the temperature to reach a final denaturation step at 95°C. All samples were normalized to *Actin7* (F01_transcript_63797) to analyze the level of expression of the candidate genes. The relative level of gene expression was calculated using the 2^–ΔΔCt^ method (Livak and Schmittgen, 2001). **Supplementary Table 2** provides a list of all the primers used in this study.

## 3 Results

### 3.1 Total protein extraction and detection

Total protein was isolated from 12 samples collected from four trees of different ages, and the protein quality of UG11Y-1 did not meet the requirements for subsequent experiments. The molecular weights of all the proteins ranged from 20–220 kDa, which indicated that the proteins did not degrade (**Supplementary Figure 1**). Although the overall protein content patterns among the 11 samples were similar, slight variations were observed among the individual samples. The total amount of protein was ≥400 µg, and the protein bands were clear, intact, and uniform characteristics without any signs of degradation. Thus, they met the requirements of the subsequent iTRAQ quantitative experiment.

### 3.2 Protein identification and analysis based on iTRAQ

In total, 537,033 total spectra, 71,247 spectra, 8,751 unique spectra, 19,246 unique peptides, 24,504 peptides, and 5,236 proteins were identified (**Supplementary Figure 2A**). Analysis of the peptide characteristics revealed that 3,998 (76.36%) proteins were related to at least two unique peptides (**Supplementary Figure 2B**). The molecular weights (MWs) of the proteins ranged from 5.2 kDa to 290.2 kDa, and the proteins within the range of 10–100 kDa accounted for 89.90% (4,707) of the total proteins (**Supplementary Figure 2C**). The average length of the identified peptides was 12.84 amino acids, which was within a reasonable range (**Supplementary Figure 2D**). These results indicated that the protein detection process was highly reliable.

### 3.3 Protein functional enrichment analysis

The identified proteins were functionally annotated using the GO, KEGG, NR, eggNOG, and COG databases. We annotated 5,234 proteins, including 4,581 in GO, 2,853 in KEGG, 2,680 in COG, 5,233 in NR, and 5,132 in eggNOG. Blast2GO software was used to compare all identified proteins with the NR database and obtain the corresponding GO functional annotations. For biological processes, the annotation results indicated that 2,470 and 2,336 proteins were significantly enriched in metabolic processes and cellular processes, respectively. With respect to cell components, 2,598 and 2,583 proteins were enriched in cells and parts of cells, respectively. By conducting the molecular function analysis, we found that 2,450 identified proteins were enriched in catalytic activity, whereas 2,059 identified proteins showed binding activity; the binding involved DNA/RNA/ATP/GTP/metal ions (**Supplementary Table 3**).

Pathway-based analysis was performed to determine the biological functions of the proteins more comprehensively. The results of the KEGG pathway analysis revealed that out of the total number of functional annotations (1,666), 119 involved different types of metabolic pathways (**Supplementary Table 4**). Carbon metabolism (ko01200) had the greatest number of protein annotations (162 annotations), followed by amino acid biosynthesis (ko01230) (146 annotations), endoplasmic reticulum protein processing (ko04141) (131 annotations), and ribosomes (ko03010) (124 annotations).

### 3.4 iTRAQ quantitative analysis

The analysis of the abundance of proteins in the immature xylem of *E. urograndis* of different ages revealed that the high-abundance proteins at UG3Y were mainly concentrated in translation, ribosome structure, and biogenesis (J) in the COG, KOG, and NOG databases. These proteins were identified as ribosomal proteins in the Pfam and SwissProt databases and were enriched in the ribosome (ko03010) pathway according to the KEGG database. We speculated that the immature xylem of *E. urograndis* has high ribosome activity when the plant is three years old. The high-abundance proteins at UG6Y, UG9Y, and UG11Y were abundant in terms of posttranslational modifications, protein turnover, and chaperones (O) (**Supplementary Table 5**).

**Figure 1:**
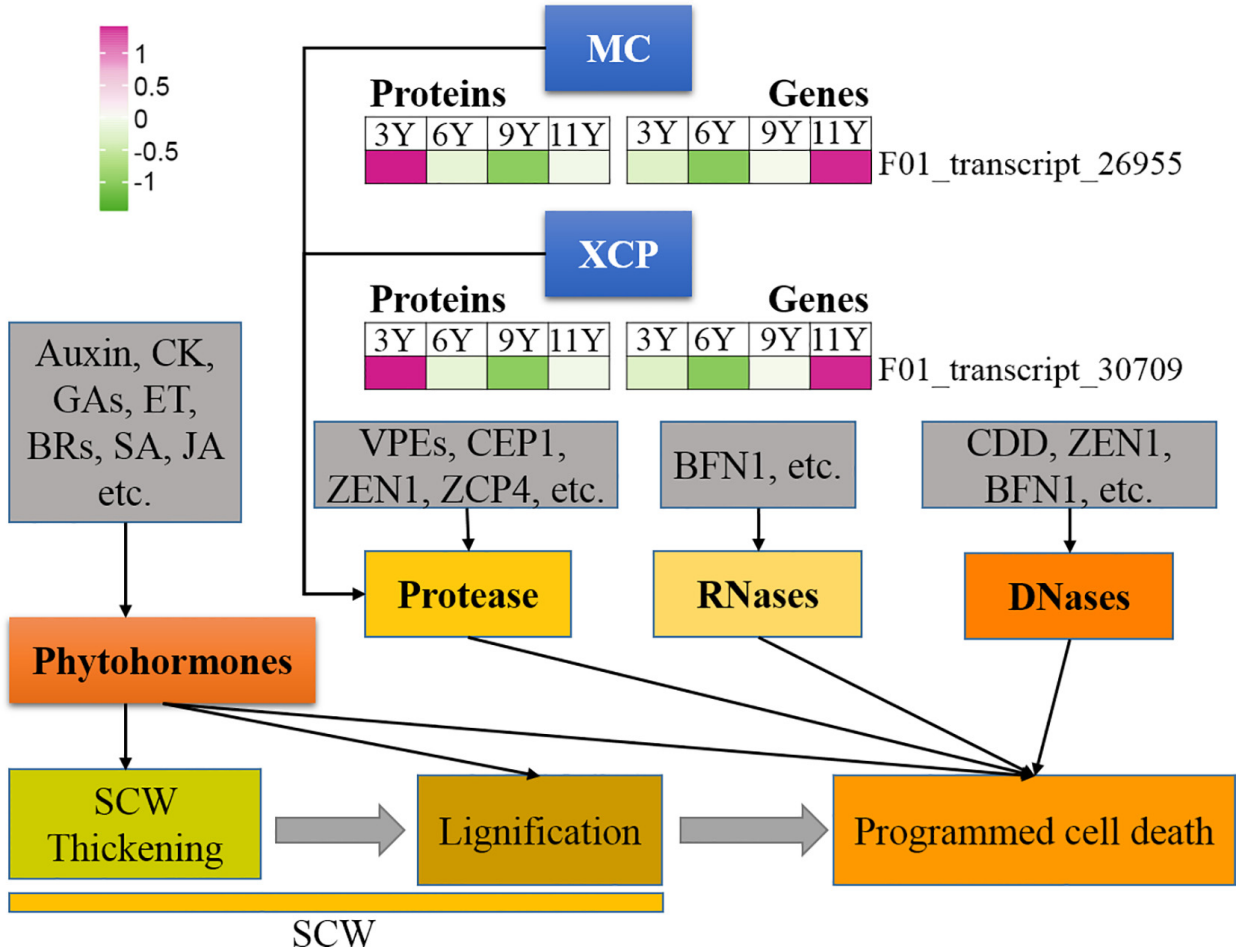
Expression profile of the DAPs (left) and their corresponding coding genes (right) involved in programmed cell death. BFN1, Bifunctional nuclease 1; BRs, Brassinosteroids; CDD, Ca^2+^-dependent DNases; CEP1, cysteine protease 1; CK, Cytokinin; ET, Ethylene; GAs, Gibberellins; JA, Jjasmonic acid; MC, Metacaspase; SA, Salicylic acid; SCW, Secondary cell wall; VPEs, vacuolar processing enzymes; XCP, Xylem cysteine protease; ZCP4, Zinnia cysteine protease 4; ZEN1, Zinnia edonuclease 1.

Based on the threshold set for screening the differential expression of proteins (*P* value = 0.05, FC = 1.2), we analyzed the protein abundance values of the immature xylem of *E. urograndis* at different ages. In total, 492 differentially abundant proteins (DAPs) were identified (**Figure 1**). The number of DAPs in the UG3Y-vs-UG9Y comparison was the greatest (total: 301, including 208 upregulated and 93 downregulated). Second, for UG3Y-vs-UG11Y (total: 101; 55 upregulated and 46 downregulated) and UG6Y-vs-UG9Y (total: 100; 74 upregulated and 26 downregulated), the number of DAPs in UG6Y-vs-UG11Y was at least 14 (eight upregulated and six downregulated). Except for the UG3Y-vs-UG6Y comparison, the number of upregulated DAPs was lower than that of downregulated DAPs, whereas in the other comparison groups, the number of upregulated DAPs exceeded that of downregulated DAPs. These results indicated that the expression of most DAPs in the immature xylem of *E. urograndis* increased with tree age.

### 3.5 Enrichment analysis of DAPs

GO functional enrichment analysis of the DAPs in the immature xylem of *E. urograndis* at four different ages revealed that 434 DAPs were enriched in 737 terms across the six comparison groups. The UG3Y-vs-UG9Y comparison group presented the greatest number of DAPs, with a total of 266 DAPs enriched across 506 GO terms (**Supplementary Table 6**). Among the 737 terms associated with GO annotation, the term “integral component of membrane” (GO:0016021) presented the greatest number of enriched DAPs in each comparison group. For biological processes, the greatest number of DAPs were associated with oxidation-reduction processes (GO:0055114). In terms of molecular function, the greatest number of DAPs was associated with ATP binding (GO:0005524).

The KEGG pathway was considered a unified entity, and a hypergeometric test was performed to identify significantly enriched pathways for the DAPs. In this study, a total of 182 DAPs were enriched in 82 pathways (**Supplementary Table 7**). In the UG3Y-vs-UG11Y comparison group, which represents the contrast between young and mature forests, a total of 41 DAPs were enriched in 37 KEGG pathways. These pathways primarily included protein processing in the endoplasmic reticulum (ko04141, 17), phenylalanine metabolism (ko00360, 4), and phenylpropanoid biosynthesis (ko00940, 4). The DAPs in the remaining 5 comparison groups primarily participated in glycolysis/gluconeogenesis (ko00010), carbon metabolism (ko01200), biosynthesis of amino acids (ko01230), ribosomes (ko03010), and other pathways associated with plant growth and development. The phenylpropanoid biosynthesis (ko0940) pathway, closely associated with wood formation, was enriched in nine DAPs. Additionally, four DAPs were identified as being involved in cellulose synthesis within the immature xylem of *E. urograndis* at various ages.

### 3.6 Differential protein analysis during wood formation

Wood formation is a complex, multistep developmental process that occurs due to the accumulation of secondary xylem. The secondary growth of trees strongly influences wood yield; thus, it is extremely valuable for research. The differentiation of cells in the secondary xylem can be categorized into four stages: cell division and differentiation, cell expansion, thickening of the SCW, and lignification of the cell wall. However, no distinct demarcation is found between stages.

#### 3.6.1 Cell division and differentiation

**Figure 2:**
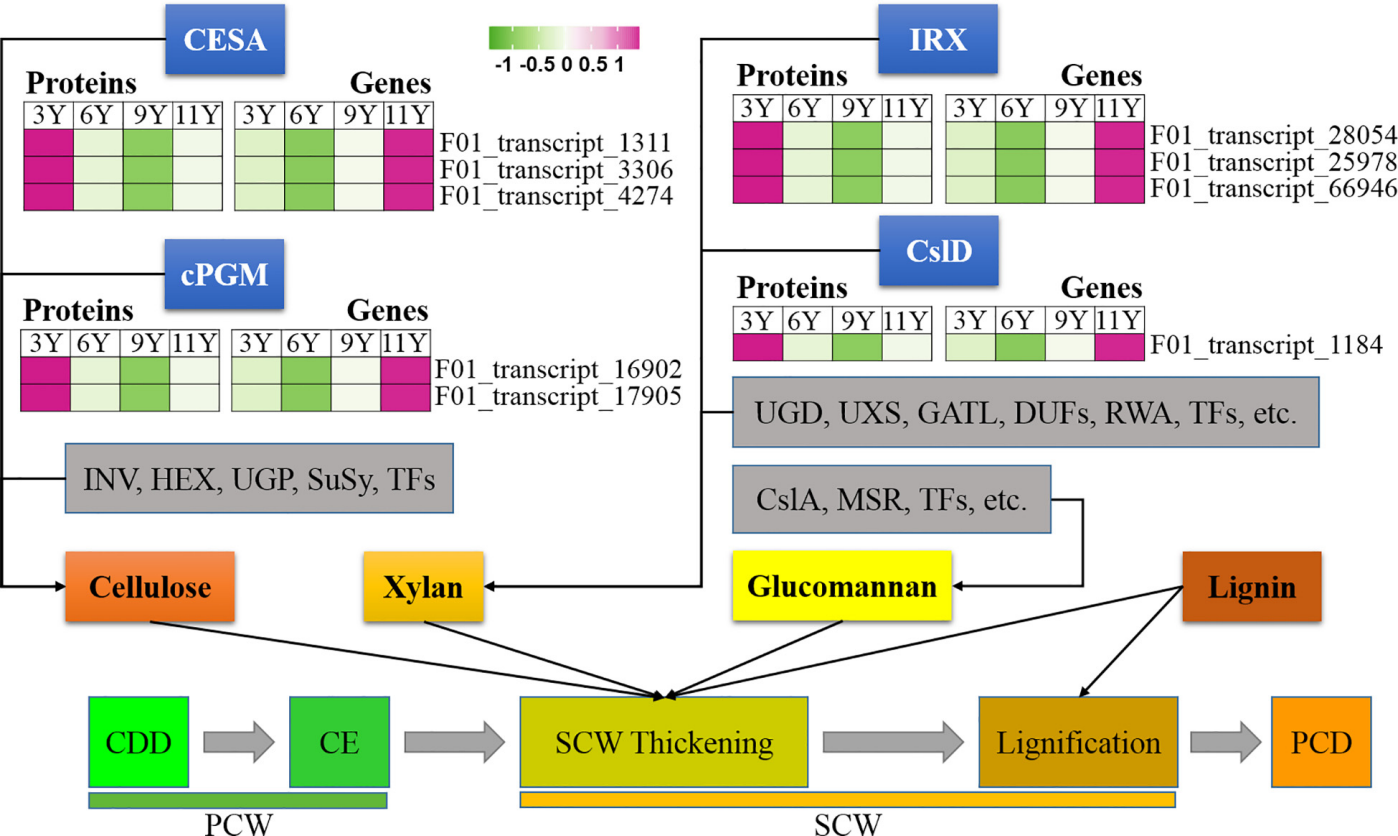
Expression profiles of the DAPs (left) and their corresponding coding genes (right) involved in secondary cell wall thickening. CESA, Cellulose synthase; cPGM,; INV, invertase; HEX, hexokinase; UGP, UDP-glucose pyrophosphorylase; SuSy, Sucrose Synthase; IRX, irregular xylem; Csl, Cellulose synthase like; UGD, UDP-glucose dehydrogenase; UXS, UDP-xylose synthase; GATL, galacturonosyl transferase-like; DUF, domain of unknown function; RWA, reduced wall acetylation; MSR, MANNAN-SYNTHESIS RELATED; CDD, Cell division and differentiation; CE, Cell expansion; PCD, Programmed cell death; PCW, Primary cell wall; SCW, Secondary cell wall.

In this study, we conducted an analysis of various proteins present in the immature xylem of *E. urograndis* at different stages of cell division and differentiation (**Figure 2**). Significant differences were observed in the abundance of two CDC proteins closely associated with cell division and differentiation, namely, F01_transcript_46705 and F01_transcript_99127. The expression level of F01_transcript_46705 was greater in the immature xylem of three-year-old eucalyptus trees than in that of transcript_99127, whereas the protein abundance of F01_transcript_99127 was greater in older eucalyptus trees. qRT-PCR analysis confirmed that the gene expression patterns corresponding to these two CDC proteins mirrored their protein abundance levels, with a higher expression level for F01_transcript_46705 observed in younger trees and a higher expression level for F01_transcript_99127 observed in older trees. Histone H2A (F01_transcript_36038) and its encoding gene were downregulated in immature xylem samples from UG11Y. Interestingly, the changes observed in the abundance levels of the kinesin proteins (F01_transcript_2952, F01_transcript_3305, and F01_transcript_97032) contrasted with their respective gene expression patterns. These findings suggest that posttranscriptional regulation (PTR) or posttranslational modifications (PTMs) may occur for the three kinesins detected within the immature xylem samples from *E. urograndis* at four different ages. Additionally, two CDK proteins (F01_transcript_18561 and F01_transcript_110872) were identified, and their abundance decreased with the age of the trees (**Figure 2**). However, there were significant differences in the expression patterns of their respective coding genes. The expression pattern of the F01_transcript_110872 gene exhibited a concordant trend with its protein abundance changes, whereas conversely, the expression pattern of the *CDKD3* gene (F01_transcript_18561) demonstrated an inverse correlation with its protein abundance alterations. These findings suggest that CDKD3 may play a regulatory role at the PTR or PTM level in the immature xylem of *E. urograndis* at different ages. Moreover, small peptides such as CLV, CLE, and PXY play crucial roles in regulating cell division and differentiation; however, no relevant DAPs were identified in this study.

Phytohormones regulate cell division and differentiation; among them, auxin plays a crucial role in controlling the proliferation of meristematic tissues (Rüscher et al., 2021). We identified auxin response factor (ARF) proteins, namely, F01_transcript_4888, F01_transcript_23386, and F01_transcript_86312 (**Figure 2**). The abundance of these three ARF proteins within the immature xylem varied significantly across different ages. In UG9Y, the expression of F01_transcript_4888 was greater than that of the other two proteins. Conversely, in UG3Y, the expression levels of F01_transcript_23386 and F01_transcript_86312 were greater than that of F01_transcript_4888. The qRT-PCR assays demonstrated synchronized changes in the expression of these three *ARF* genes and their corresponding protein abundances, indicating that functional variations among different ARF proteins exhibited functional variation in the immature xylem of *E. urograndis* at various ages. However, no DAPs were identified in this study for other plant hormones, including CK, BRs, GAs, and ABA.

#### 3.6.2 Cell expansion

**Figure 3:**
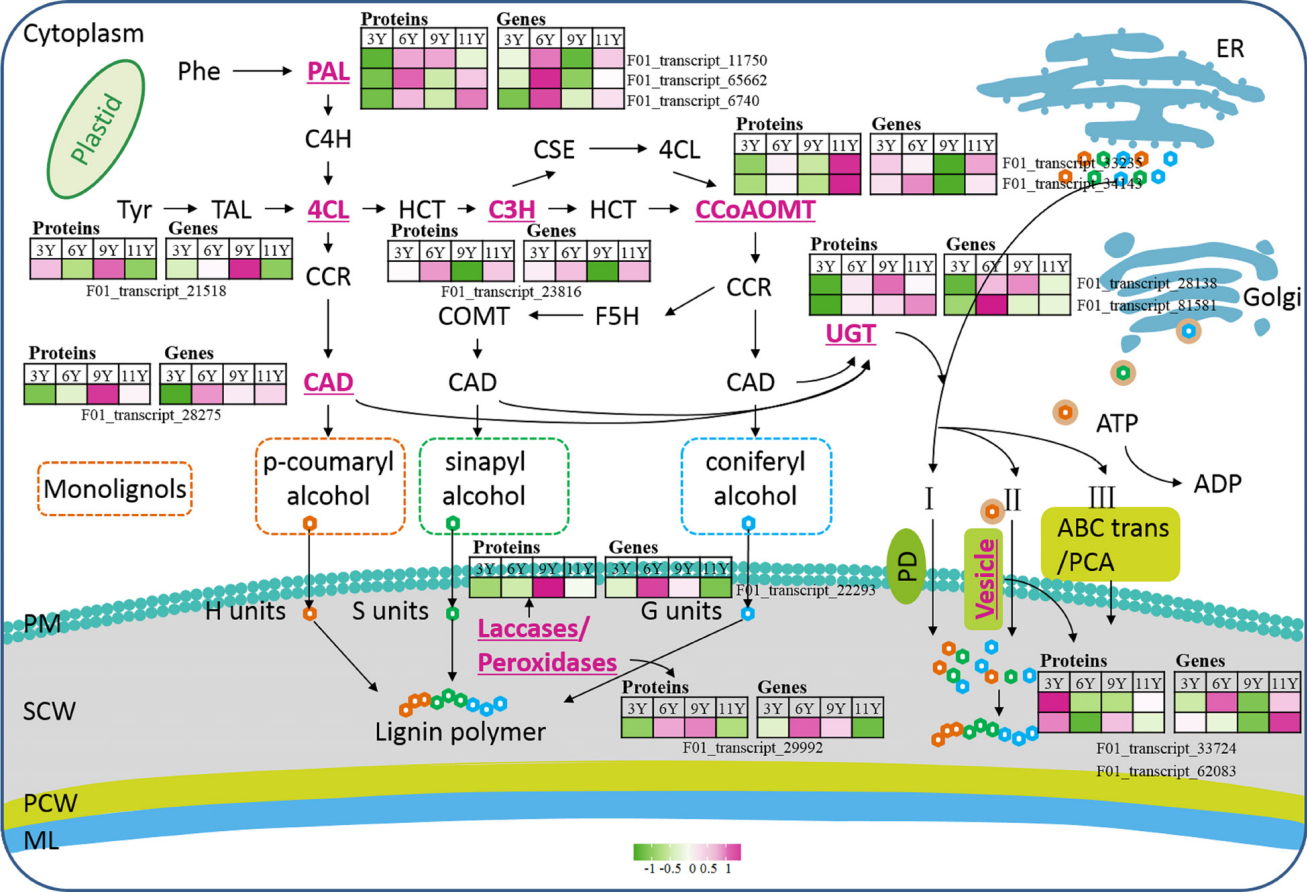
The main process of plant lignification. Phe, Phenylalanine; PAL, Phenylalanine ammonia lyase; C4H, cinnamate 4-hydroxylase; 4CL, 4-coumaric acid: coenzyme a ligase; Tyr, Tryptophan; TAL, Tyrosine ammonia lyase; HCT, Hydroxycinnamoyl tranferase; C3H, Coumarate 3-hydroxylase; CSE, caffeoyl shikimate esterase; CCoAOMT, Caffeoyl-CoA O-methyltransferase; CCR, Cinnamoyl CoA reductase; CAD, Cinnamyl alcohol dehydrogenase; F5H, Ferulate 5-hydroxylase; COMT, Catechol-O-methyltransferase; UGT, UDP-glycosyltransferases; PCA, Proton coupled antiporters; PD, Passive diffusion; PM, Plasma membrane; SCW, Secondary cell wall; PCW, Primary cell wall; ML, Middle lamella.

Luo and Li (2022) demonstrated that the expansion of xylem cells during wood formation is regulated by a variety of factors, including plant hormones, expansins, hydrolases, and other molecular networks. Expansins (EXPs) play crucial roles in promoting cell wall extension during plant growth (Kök et al., 2023). In this study, we observed a decrease in the abundance of UG11Y for both EXPA proteins (**Figure 3**). However, the level of abundance of the EXPA2 protein (F01_transcript_116943) was greater in UG6Y and UG9Y. Additionally, the expression level of its encoding gene *EXPA2* was greater in UG3Y and UG6Y. Similarly, we found that the abundance of the EXPA13 protein (F01_transcript_29263) was greater in UG3Y, with its expression level being greater in UG6Y and UG9Y. Therefore, our conclusion is that PTR or PTMs occur for both the EXPA2 and EXPA13 genes within immature xylem tissues of *E. urograndis* at different ages. However, other proteins associated with cell expansion, such as KNAT7, growth-regulating factor 4 (GRF4), and other TFs, were not identified as DAPs in this study.

Xyloglucan hydrolase (XTH) enzymes participate in the loosening and remodeling of cell walls, potentially by catalyzing the hydrolysis and reconnection of xyloglucan (Xie et al., 2015). Our findings indicate that the XTH27 protein (F01_transcript_77387) exhibited differential abundance and was significantly upregulated in UG3Y cells (**Figure 3**). However, its expression pattern contrasted with that of the *XTH27* gene, while *XTH27* gene expression was also upregulated in UG11Y. These findings suggest that PTR or PTMs may influence the expression of XTH27 across different ages in the immature xylem of *E. urograndis*.

In addition to EXPs and XTH, glycosyl hydrolases such as endo-1,4-β-glucanases (EGases) and endo-1,4-β-mannanase (MAN), participate in cell wall loosening (Yu et al., 2013; Zhao et al., 2013). We identified four EGases (F01_transcript_88135, F01_transcript_32721, F01_transcript_33626, and F01_transcript_34728) and one EgMAN7 (F01_transcript_62437) in the immature xylem of *E. urograndis* (**Figure 3**). The abundance of the four EGases was greater in UG11Y, whereas that of EgMAN7 was greater in UG9Y. The qRT-PCR assays revealed synchronized changes between the abundance levels of F01_transcript_88135 and F01_transcript_33626 and their corresponding gene expression levels. Additionally, upregulation of two *EGase* genes (F01_transcript_32721 and F01_transcript_34728) occurred in UG9Y, suggesting PTR or PTMs within the cells of immature xylem across UG3Y and UG9Y. However, upregulation of the *EgMAN7* gene was observed in UG11Y, indicating PTR or PTMs across all four age groups.

Pectin, a primary constituent of the cell wall, plays a crucial role in the remodeling and expansion processes of the cell wall (Shin et al., 2021). In this study, we observed significant downregulation of three pectinesterase 3 genes (PE3, F01_transcript_21425, F01_transcript_21735, F01_transcript_63492) in UG11Y but upregulation in UG9Y (**Figure 3**). The expression patterns of these three genes were significantly different. Specifically, F01_transcript_21425 was expressed at higher levels in UG3Y but at lower levels in UG6Y. On the other hand, both UG9Y and UG11Y presented increased expression levels of F01_transcript_21735. Furthermore, the expression level of F01_transcript_63492 was greater in both UG6Y and UG9Y. To summarize our findings, the regulation of two pectinesterase genes (F01_transcript_21425 and F01_transcript_63492) in the immature xylem of *E. urograndis* at four different ages occurs at the PTR or PTM level, whereas F01_transcript_21735 is regulated at the PTR or PTM level in 6–11-year-old trees. Additionally, there was a positive correlation between the abundance pattern of pectin methyltransferase protein (PME, F01_transcript_103117) and its corresponding gene expression pattern. Higher expression levels were detected in individuals aged three and six years than in those aged nine and eleven years.

Phytohormone signals may play pivotal roles in the regulation of cell elongation and expansion (Luo and Li, 2022). GAs and ET have been reported to be closely associated with the process of cell expansion (Eriksson et al., 2000; Wessels et al., 2019). In this study, we observed a decrease in the expression levels of ethylene receptor-like protein (ERF), represented by F01_transcript_4077, as well as ethylene-insensitive protein 2 (EIN2), represented by F01_transcript_71448, in UG11Y (**Figure 3**). The results obtained from qRT-PCR analysis revealed significant differences in the expression patterns of these two genes. *ERF* presented relatively high expression levels in UG9Y and UG11Y, whereas *EIN2* presented increased expression in UG6Y and UG9Y. It is speculated that PTR or PTMs may occur for ERFs during tree growth at the 3-, 9-, and 11-year-old stages. Similarly, PTR or PTMs might affect EIN2 during tree growth at the 3-, 6-, and 9-year-old stages.

#### 3.6.3 Secondary cell wall thickening

**Figure 4:**
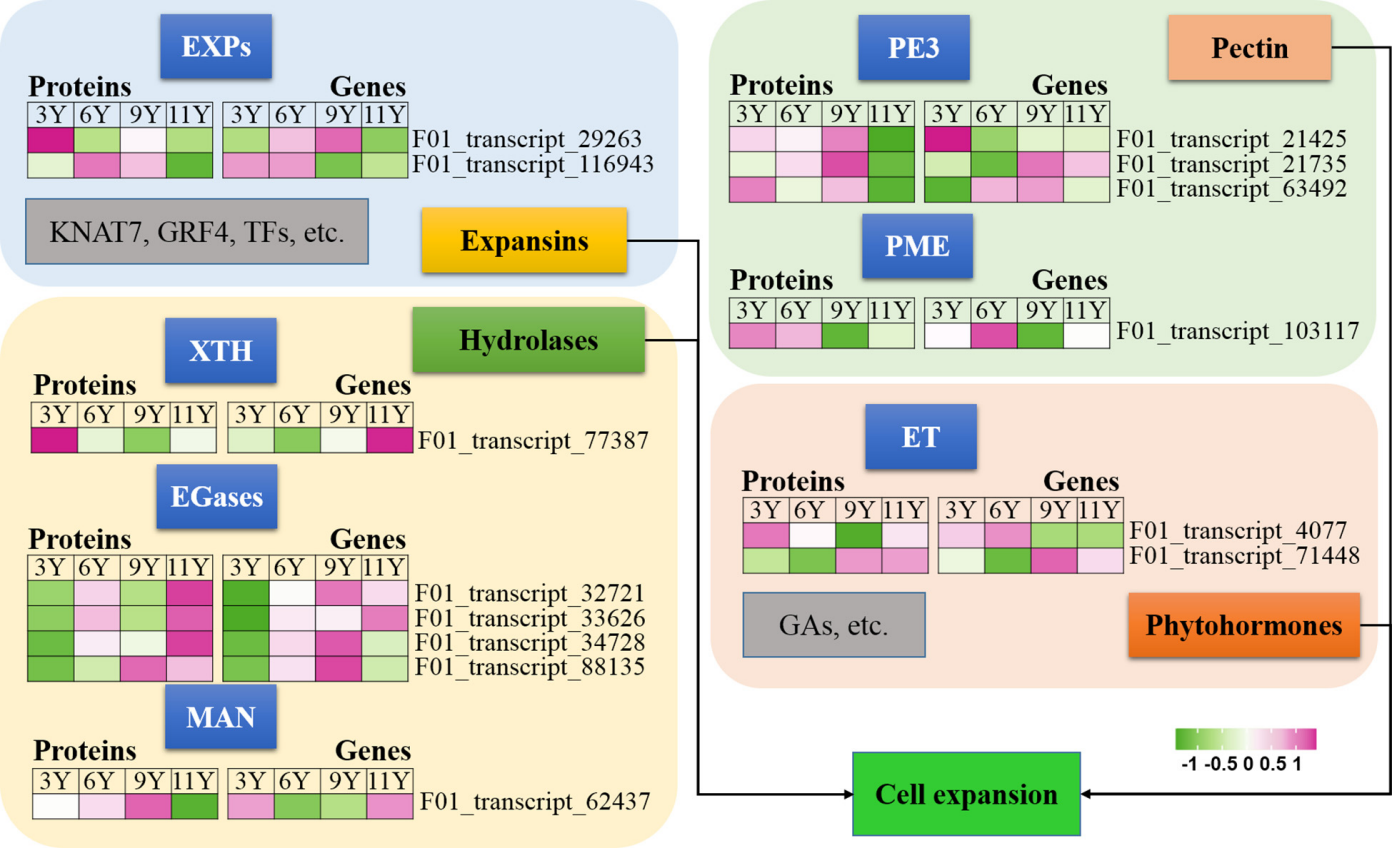
Expression profiles of the DAPs (left) and their corresponding coding genes (right) involved in cell expansion. EGases, endo-1,4-β-glucanases; ET, Ethylene; EXPs, EXPANSINs; GAs, Gibberellins; GRF4, Growth-Regulating Factor 4; KNAT7, KNOTTED ARABIDOPSIS THALIANA7; XTH, Xyloglucan hydrolase; MAN, endo-1,4-β-mannanase; PE3, Pectinesterase 3; PME, Pectin methyltransferase protein.

The thickening of the SCW initiates after cell expansion (Luo and Li, 2022). Through the analysis of 5,236 proteins derived from the immature xylem of *E. urograndis* at four different ages, we observed a relatively high abundance of five CesA proteins in UG3Y (**Figure 4**). These included three DAPs: CesA3 (F01_transcript_1311), CesA4 (F01_transcript_3306), and CesA8 (F01_transcript_4274). qRT-PCR confirmed that all three *CesA* genes were upregulated in UG3Y. The expression patterns of *CesA4* and *CesA8* generally correlated with changes in their corresponding protein abundances. However, there were variations between the expression patterns of *CesA3* and its associated proteins among UG6Y, UG9Y, and UG11Y. These differences are likely attributed to PTR or PTMs at these specific ages.

In plant cells, cytoplasmic glucose phosphate mutase (cPGM) plays a crucial role in photosynthesis, respiration, and cell wall biosynthesis by facilitating the interconversion between glucose-1-phosphate and glucose-6-phosphate (Li et al., 2015). In this study, we identified two cPGM proteins (F01_transcript_16902 and F01_transcript_17905) that were upregulated in UG9Y and UG11Y (**Figure 4**). The results of the qRT-PCR analysis revealed synchronized changes in the expression of F01_transcript_17905 with that of its corresponding gene. Conversely, while the gene expression of F01_transcript_16902 was greater in UG3Y, its encoded protein displayed the opposite pattern. Therefore, we infer that PTR or PTMs occur for F01_transcript_16902 in the immature xylem of *E. urograndis* at different ages. The differences in the abundances of other proteins closely associated with cellulose biosynthesis, such as INV, HEX, UGP, and Susy, were not significant at different ages.

Unlike PCW, SCW contains xylan instead of xyloglucan (Wang et al., 2016). Studies have demonstrated that IRX10 and IRX15L are key players in xylan biosynthesis (Brown et al., 2011; Wang et al., 2022). In this study, one IRX15L protein (F01_transcript_28054) and two IRX10 proteins (F01_transcript_25978 and F01_transcript_66946), closely associated with xylan biosynthesis, were significantly upregulated in UG3Y but decreased over time (**Figure 4**). The expression patterns of F01_transcript_25978 and its corresponding gene remained consistent across different ages, whereas F01_transcript_28054 and its encoded protein were upregulated in UG3Y. However, the expression level changes in trees aged 6, 9, and 11 years did not correspond with the observed variations in their encoded protein translation levels. These findings suggest that PTR or PTMs may occur during these ages. Similarly, the expression pattern of the gene encoding F01_transcript_66946 differed from its corresponding protein abundance in trees aged between 6 and 11 years because of potential PTRs or PTMs at these different ages. Additionally, one CslD3 protein (F01_transcript_1184) involved in hemicellulose biosynthesis was significantly upregulated along with its encoded gene in UG3Y.

#### 3.6.4 Lignification

**Figure 5:**
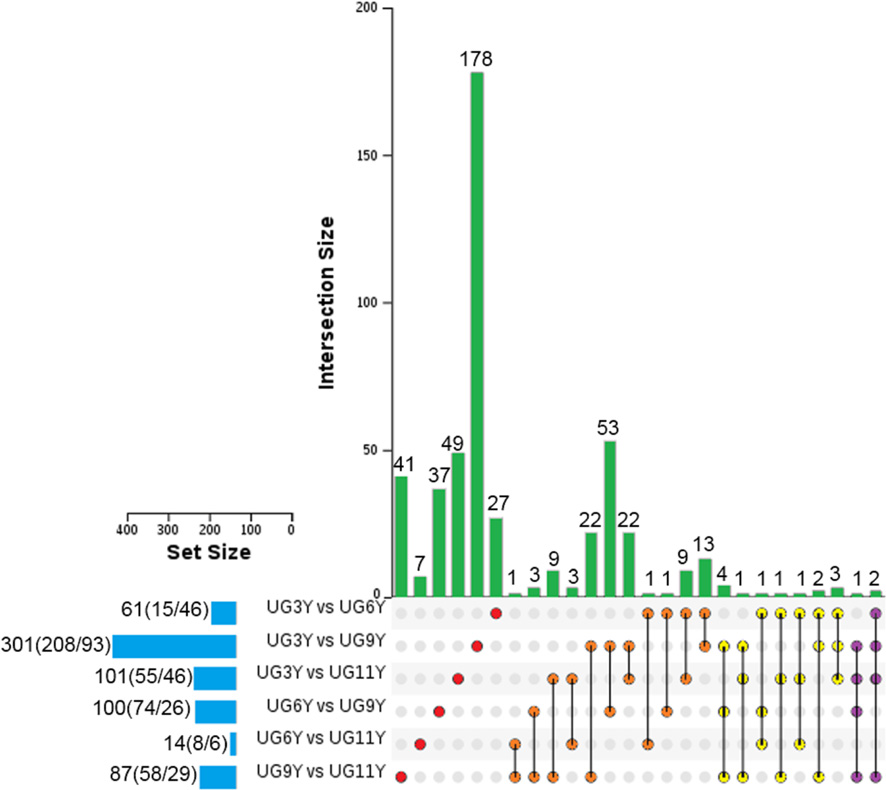
UpSet of DAPs between pairwise comparisons of any two samples from the vascular cambium of *E. urograndis at different ages*. The horizontal bar chart on the left shows the number of DAPs in various comparable groups (upregulated/downregulated), the lines between points represent the intersection of various comparable groups, and the vertical bar chart represents the corresponding number of intersection comparable groups.

Lignin is synthesized and deposited on the cell wall through lignification. This process primarily involves three developmental stages: the biosynthesis of lignin monomers in the cytoplasm, the transport of lignin monomers across the cell membrane, and the deposition of lignin monomers through oxidative polymerization on the cell wall (Guo et al., 2020) (**Figure 5**). In this study, among the nine DAPs closely associated with lignin monomer biosynthesis, two caffeoyl-CoA O-methyltransferase (CCoAMT) proteins (F01_transcript_33235 and F01_transcript_34143) were upregulated in UG11Y (**Figure 5**). The changes in the protein abundance of F01_transcript_33235 were synchronized with its encoding gene expression pattern. However, there was a notable disparity between changes in the protein abundance of F01_transcript_34143 and its encoding gene expression; its level of expression was greater in UG6Y. Therefore, it can be inferred that PTR or PTMs may influence the expression of F01_transcript_34143 at different ages in *E. urograndis*. The expression levels of C3’H (coumarate 3-hydroxylase) protein (F01_transcript_23816) and its encoding gene were downregulated in UG9Y but were highest in UG6Y. The proteins 4CL (4-coumaric acid: coenzyme a ligase, F01_transcript_21518) and CAD (cinamyl alcohol dehydrogenase, F01_transcript_28275) were upregulated in the immature xylem tissues of UG9Y. The results of the qRT-PCR assays revealed that the changes in the expression of 4CL and its coding gene were similar, whereas *CAD* exhibited a different pattern compared with the changes observed for its encoded protein abundance in UG6Y and UG9Y, likely due to PTR or PTMs occurring at these two tree ages. The expression levels of three phenylalanine ammonia lyase (PAL) proteins, namely, F01_transcript_11750, F01_transcript_65662, and F01_transcript_6740, decreased in UG3Y but increased in UG6Y. Among these proteins, F01_transcript_65662 and F01_transcript_11750 were the most abundant in UG6Y, whereas F01_transcript_6740 was the most abundant in UG11Y. The results obtained from the qRT-PCR assays indicated that the changes in the expression of the gene encoding F01_transcript_11750 were consistent with the changes observed at the protein level. The expression of F01_transcript_6740 likely also underwent PTR or PTMs in trees aged three, six, and nine years. Similarly, the expression of F01_transcript_65662 was likely influenced by PTR or PTMs in trees aged 3, 6, 9, and 11 years.

The biosynthesis of lignin monomers occurs in the cytoplasm or near the endoplasmic reticulum, and their transport across the membrane into the developing cell wall is a complex process involving multiple steps. In this study, we observed significant upregulation of two vesicle-associated membrane proteins, 721-like/722 (F01_transcript_62083 and F01_transcript_33724), in UG3Y (**Figure 5**). Interestingly, the expression patterns of these two genes differed significantly from the changes in protein abundance, suggesting potential PTR or PTMs in trees of different ages. Lignin monomers present in the cytoplasm can be transported via UDP-glycosyltransferases (UGTs) to form monolignol glucosides (Guo et al., 2020). UGTs constitute a large family of glycosyltransferase genes in plants. In this study, we identified two UGTs associated with lignin biosynthesis and transmembrane transport. Notably, UGT76E2 (F01_transcript_81581) was significantly upregulated in UG11Y and presented the highest expression level in UG6Y. Conversely, both its encoding gene and protein were downregulated in UG3Y. Additionally, while UGT76F1 (F01_transcript_28138) was upregulated in UG9Y, it was downregulated specifically in UG3Y. These findings suggest that PTR or PTMs may occur for both UGT76E2 and UGT76F1, specifically between the ages of 6 and 11 years, in *E. urograndis*.

The movement of the lignin monomer within the cell is unrestricted, while the fixed oxidase restricts its mobility and determines the specific location for lignin polymer formation. Previous studies have demonstrated that plant laccases (LAC) and type III peroxidases (PRX) can catalyze the oxidative polymerization of lignin monomers (Schuetz et al., 2014; Shigeto and Tsutsumi, 2016). In this study, we observed an upregulation of LAC14 (F01_transcript_22293) and a peroxidase protein (F01_transcript_29992) in UG9Y (**Figure 5**). Furthermore, we found that the expression levels of these two genes were greater in UG6Y, suggesting that potential PTR or PTMs may have occurred in LAC14 and PRX across four distinct ages.

#### 3.6.5 Programmed cell death

**Figure 6:**
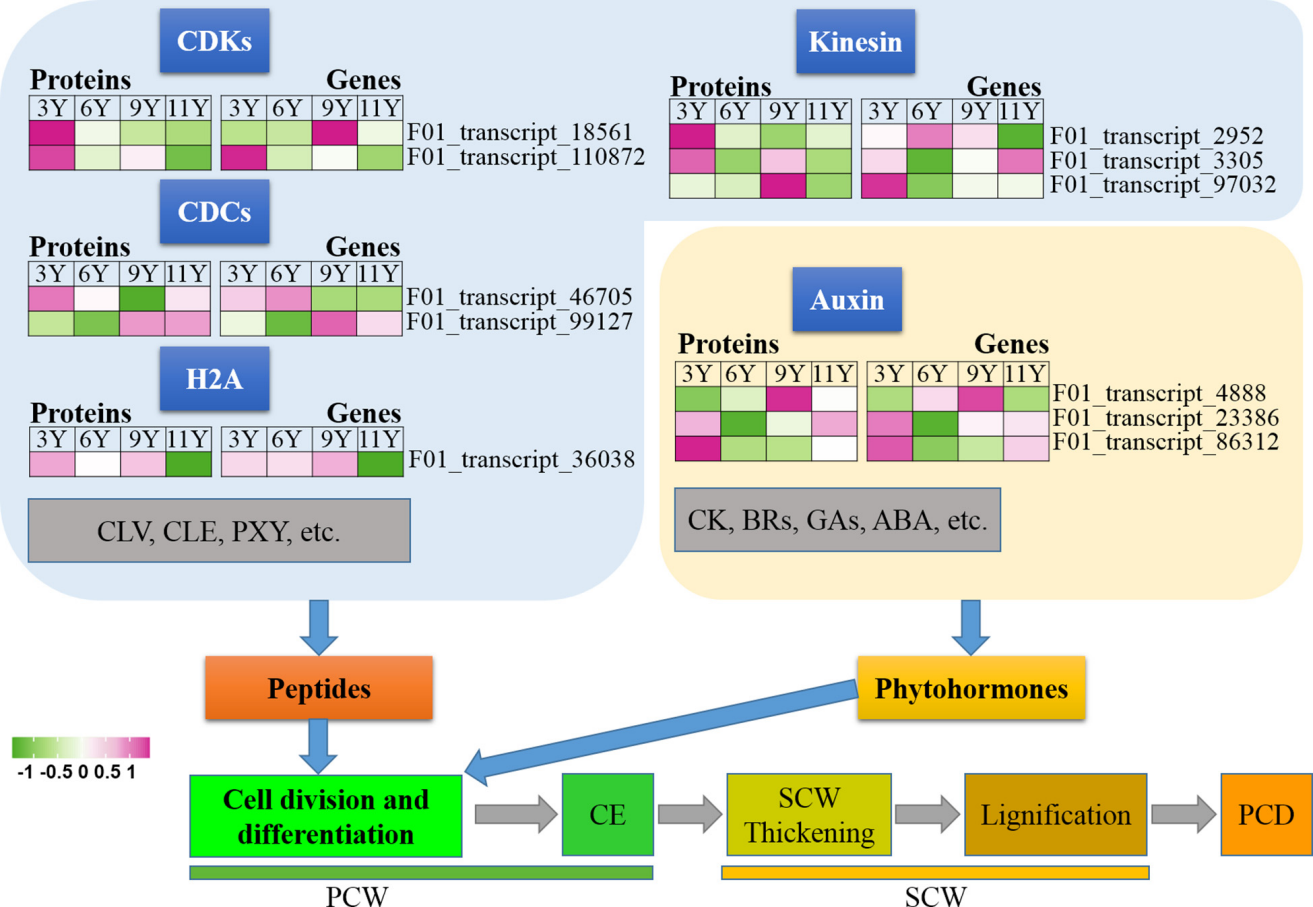
Expression profiles of the DAPs (left) and their corresponding coding genes (right) involved in cell division and differentiation. ABA, Abscisic acid; BRs, Brassinosteroids; CDCs, cell division cycle proteins; CDKs, Cyclin-dependent kinases; CE, Cell expansion; CK, Cytokinin; CLE, CLAVATA3/ESR; CLV, CLAVATA; ETH, Ethylene; GAs, Gibberellins; H2A, Histone 2A; PCD, Programmed cell death; PCW, Primary cell wall; PXY, Phloem intercalated with xylem; SCW, Secondary cell wall.

Like caspases in animal systems, metacaspase (MC) in plants is a type of cysteine protease that functions as the primary executor of PCD in plants (Li et al., 2008). In this study, we identified a METACASPASE 4-like (MC4-like) strain (F01_transcript_26955), and its abundance increased with tree age (**Figure 6**). Therefore, it can be speculated that the activity of this protein may increase in immature xylem as trees mature. The results from the qRT-PCR assay revealed that the *MC4-like* expression level was the highest in UG9Y because PTR or PTMs occurred specifically in immature xylem across different tree ages. Previous studies have indicated that XYLEM CYSTEINE PROTEASE 1 (XCP1) and XCP2 proteins are involved in catheter differentiation as autophagy effectors in *Arabidopsis* (Avci et al., 2008). In this study, the protein XCP2 (F01_transcript_30709) presented the highest abundance in UG11Y, while its gene expression level was also highest at this age. However, both protein abundance and coding gene expression were significantly lower in UG3Y than in the other age groups. These findings suggest that PTR or PTMs might occur for XCP2 in three- and nine-year-old trees. Additionally, the levels of PCD-associated proteases such as ZCP4 (Zinnia cysteine protease 4) and CEP1 (Cysteine protease 1), as well as nucleases such as BFN1 (Bifunctional nuclease 1, both DNase and RNase), and ZEN1 (Zinnia endonuclease 1), remained relatively stable in the immature xylem of *E. urograndis* across different developmental stages. The involvement of plant hormones in plant development PCD has been reported; however, no DAPs associated with plant hormone signaling were identified in this study.

## 4 Discussion

The difference in wood yield between young and adult trees is prominent for most tree species. As tree age increases, there is a decrease in the continuous division of vascular cambium cells, resulting in a shortened duration and decreased wood yield. Additionally, there are variations in both the number and expression abundance of translated proteins with increasing tree age. Celedon et al. (2007) analyzed DAPs in the cambium region of *E. grandis* at three different ages: six-month-old seedlings, three-year-old trees, and six-year-old trees. These findings provide valuable information about the proteome of *E. grandis* during wood formation in juvenile plants. Similarly, Zhang et al. (2021) conducted a transcriptomic and proteomic analysis of the stem xylem at different stages (1 month, 2 months, and 3 months) of *P. tomentosa* development, revealing the potential extensive involvement of PTR in the development of lignification. To address the lack of proteomic information during the developmental stages from juveniles to maturity, we generated a protein accumulation dataset for *E. urograndis*, which is primarily used as a tree species for eucalyptus plantations because of its adaptability and high yield potential.

We employed iTRAQ technology to conduct qualitative protein analysis of the immature xylem of *E. urograndis* at four different ages. Through 2-D-LC-MS/MS, we successfully identified 5236 proteins, which is notably 21.81 times greater than the previously reported count (240) by Celedon et al. (2007). The results of the quantitative analysis revealed that ribosome activity persisted in the immature xylem of three-year-old trees, whereas proteins associated with cell wall biosynthesis presented increased activity in six-year-old and nine-year-old trees. Moreover, proteins involved in lignin biosynthesis and intercellular material transport presented increased activity, specifically in 11-year-old trees. Consequently, it can be inferred that during the three-year stage, E*. urograndis*’ immature xylem cells primarily undergo cell division and differentiation processes. A substantial amount of SCW is subsequently synthesized during the critical growth phase when the trees reach six and nine years of age for diameter expansion purposes. Finally, significant lignin production was observed within the 11-year-old trees as they underwent wood lignification.

The expression of DAPs in the immature xylem of *E. urograndis* at four different ages was analyzed, resulting in the identification of 492 DAPs. In a parallel study conducted by Zhang et al. (2021), proteomic analysis of stem xylem from *P. tomentosa* at three different ages (1, 2, and 3 months) revealed a total of 445 DAPs. Notably, comparative analysis between these two significant fast-growing tree species demonstrated a substantial degree of functional similarity on the basis of GO annotation. The enrichment analysis results indicated that these DAPs were associated primarily with posttranslational modification, protein turnover, chaperone (O), carbohydrate transport and metabolism (G), translation, ribosomal structure and biogenesis (J), and energy production and conversion (C). These findings suggest that efficient cell proliferation during age-related changes necessitates high energy metabolism, the regulation of carbohydrate and amino acid metabolism, and PTMs for self-regulatory responses. An analysis focusing on these wood formation-associated DAPs may provide a solid theoretical foundation for further understanding the underlying molecular mechanisms involved.

In this study, we examined the expression patterns of DAPs and their corresponding coding genes associated with wood formation in the immature xylem of *E. urograndis* at four different ages. DAPs involved in early stages of wood formation, including cell division and differentiation, presented relatively high levels of expression in the immature xylem of three-year-old trees. This observation suggests an increase in cell division and differentiation processes in younger trees. The expression profiles of protein families such as CDCs, CDKs, and ARFs in the immature xylem vary significantly across different ages, indicating their involvement in diverse biological functions. Furthermore, three kinesin genes (F01_transcript_2952, F01_transcript_3305, and F01_transcript_97032), along with CDKD3 (F01_transcript_18561), were found to be regulated by both PTR and PTMs within the immature xylem of trees at four different ages. The findings presented here are consistent with those of previous studies conducted by Chowdhury et al. (2021) and Iyer et al. (2022), which demonstrated that the regulation of the kinesin-like proteins NACK2 and CDK-2 may be influenced by PTR.

During cell division and differentiation, as well as wood-induced cell expansion, the primary component of the cell wall is the PCW. Cell expansion mainly occurs in both the radial and axial directions. Throughout this process, a majority of the DAPs presented relatively high expression levels in the immature xylem of three-, six-, and nine-year-old trees. Our findings suggest that continuous cell growth takes place in the immature xylem at these ages. Queiroz de Pinho Tavares et al. (2020) demonstrated that miRNAs can specifically target genes involved in processes such as cell expansion, cell separation, hemicellulose metabolism, and cellulose hydrolysis, including *ExpA7*, *PE*, *XTH*, and *EGases*. These findings align with our findings indicating that genes encoding proteins such as EXPA2, EXPA13, XTH27, and EGases are regulated by PTR. We also discovered potential PTR or PTMs occurring at specific ages for ethylene receptor-like (F01_transcript_4077) and ethylene-insensitive protein 2 (F01_transcript_71448), as reported by Ma and Dong (2021), who reported phosphorylation events on EIN2 and ETR at the translational level. Additionally, with respect to the identification of cell expansion-related differential proteins in the immature xylem of *E. urograndis* at four different ages, certain protein-coding genes (such as *EXPA2*, *EXPA13*, *XTH27*, and *EgMAN7*) may undergo PTR or PTMs. Moreover, the genes encoding EGases, EIN2, ETR and other proteins in the immature xylem of *E. urograndis* exhibit age-specific PTR or PTMs. Consequently, transcriptional regulation varies across different ages within the immature xylem owing to the diverse biological functions exhibited by various proteins at each specific age.

Many researchers have extensively investigated the transcriptional regulation of SCW thickening and lignification. The development of SCW thickening is tightly controlled by a hierarchical transcriptional network (Taylor-Teeples et al., 2015). Additionally, it has been reported that the biosynthesis of SCW may also be regulated by PTR (Wang et al., 2020). Several studies have demonstrated that genes involved in the lignin biosynthesis pathway are subject to regulation through alternative splicing, microRNAs, and long noncoding RNAs (Sun et al., 2023; Zhang et al., 2018). Bao et al. (2013) reported that about 36% of xylem-expressed genes undergo alternative splicing, particularly those associated with cell wall biosynthesis, such as glycosyl transferases and C2H2 TFs. These findings align with our study results, indicating that the UGT76E2 protein-encoding gene is regulated by PTR. Among the 49 *LAC* genes in the *Populus* genome, ptr-miRNA397a was predicted to target 29 of these genes (Lu et al., 2013). Wang et al. (2015) proposed that certain long noncoding RNAs (lncRNAs) might regulate lignin biosynthesis by influencing *LAC4* expression. These findings support our results, suggesting a potential PTR for *LAC14* (F01_transcript_22293). Chen et al. (2015) identified lncRNAs that target 16 genes involved in the biosynthetic pathways of cellulose or lignin, including *CesA3*, *UGT*, and *CCoAMT*. Our findings are consistent with their results, suggesting that *CesA3* (F01_transcript_1311), *UGT76E2* (F01_transcript_81581), and *CCoAMT* (F01_transcript_34143) may undergo PTR. Zhou et al. (2017) also identified 16 lncRNAs and 22 potential target genes (PTGs) in the phenylpropanoid pathway, which included *PAL*, *CAD*, and *CCoAMT*. The findings of their study were similar to those of *PAL* (F01_transcript_65662 and F01_transcript_6740), *CAD* (F01_transcript_28275), and *CCoAMT* (F01_transcript_34143) in this study. Similar to observations made during the cell expansion stage, we detected differences in the transcriptional regulation among genes encoding different proteins during the SCW deposition and lignification stages in the immature xylem of *E. urograndis* at different ages. This observation may be attributed to the distinct biological functions of the immature xylem of *E. urograndis* at different ages, thereby facilitating the ability of this species to sustain its rapid growth characteristics throughout different developmental stages.

After the deposition of SCW and lignification, PCD occurs as the final step during the maturation of xylem cells (Kim et al., 2022). Generally, proteases, including XCP1, XCP2, and MC9, play pivotal roles in PCD during xylem formation (Kawabe et al., 2018; Zhang et al., 2021). Furthermore, Kawabe et al. (2018) proposed that S-nitrosylation might impact the expression of XCP genes. Protein S-nitrosylation is a prevalent redox-dependent PTM of proteins. Zhang et al. (2021) reported that *PtoXCP2.1* may be regulated by PTR. Basak and Kundu (2022) reported that plant metacaspase activity is tightly regulated to prevent inadvertent activation through a combination of genetics and PTR. These findings align with our study, where we hypothesized that *MC4-like* (F01_transcript_26955) and *XCP2* (F01_transcript_30709) could be regulated by PTMs.

In summary, the abundance of proteins associated with wood formation in the immature xylem of *E. urograndis* significantly varies with tree age, particularly when young (3-year-old) and mature (9-year-old) trees are compared. The analysis of protein abundance trends and gene expression patterns suggested that 32 out of 50 DAPs or their corresponding coding genes are regulated by PTR or PTMs. This regulatory mechanism is more pronounced during the processes of cell expansion and lignification. Notably, certain proteins, such as CDC, XTH, IRX, and XCP, have been identified as potentially undergoing PTR or PTMs on the basis of their presence in both *E. urograndis* and *P. tomentosa*. This finding may be attributed to the rapid growth rate of *E. urograndis* compared with that of other tree species. However, further research is needed for confirmation.

## 5 Conclusion

The secondary xylem of trees serves as the fundamental biological foundation for wood production, and understanding its regulatory mechanisms remains a significant challenge in forest research. The process of lignification, which is essential for wood formation in woody plants, still lacks comprehensive understanding. This knowledge gap hampers the application of molecular breeding techniques for cultivating forest trees. In this study, we conducted an extensive analysis of 5236 proteins and 492 DAPs identified in immature xylem tissues from trees of *E. urograndis* at four different ages. By conducting qRT-PCR analysis, we further investigated the differential expression patterns of these proteins during various stages of wood formation. Our findings suggest that the genes encoding kinesin, CDKD3, EXPA13, EXPA2, XTH27, EGases, UGT76E2, LAC, CCoAMT, CesA3, PAL, and CAD may undergo PTR. Additionally, genes encoding EIN2, ETR, MC4-like, and XCP may undergo PTMs. This study provides valuable insights into the proteomic changes associated with wood formation in immature xylem tissues of *E. urograndis* at the molecular level and can serve as a reference for future studies on molecular breeding strategies and targeted selection approaches.

## Data Availability

The raw mass spectrometry proteomics data for co-fractionation experiments, immunoprecipitation, and total proteome analysis have been deposited to the ProteomeXchange Consortium (https://proteomecentral.proteomexchange.org) via the iProX partner repository (Ma et al., 2019; Chen et al., 2021) with the dataset identifier PXD051510.
