# Supplementary material for: Comparative proteomic analysis provides insights into wood formation in immature xylem at different ages in Eucalyptus urophylla × Eucalyptus grandis
Source: Front Plant Sci. 2024 Oct 30;15:1431164. doi: 10.3389/fpls.2024.1431164 (PMC11557400; doi:10.3389/fpls.2024.1431164)
Supplement: Supplementary file 1 [file DataSheet1.docx]

Supplementary Material

Comparative proteomic analysis provides insights into wood formation in immature xylem at different ages in *Eucalyptus urophylla* × *Eucalyptus grandis*

Guo Liu^1,2^, Guowu Zhang^1^, Zhihua Wu^1^, Xiuhua Shang^1^, Chubiao Wang^3^, Wanhong Lu^1^, Yan Lin^1^, Anying Huang^1^, Jianzhong Luo^1,2*^

^1^Research Institute of Fast-Growing Trees, Chinese Academy of Forestry, Zhanjiang, China

^2^State Key Laboratory of Tree Genetics and Breeding, Chinese Academy of Forestry, Beijing, China

^3^ College of Coastal Agriculture Sciences, Guangdong Ocean University, Zhanjiang, China

*** Correspondence:**Jianzhong Luo
LuojzEC@caf.ac.cn

# Supplementary Figures


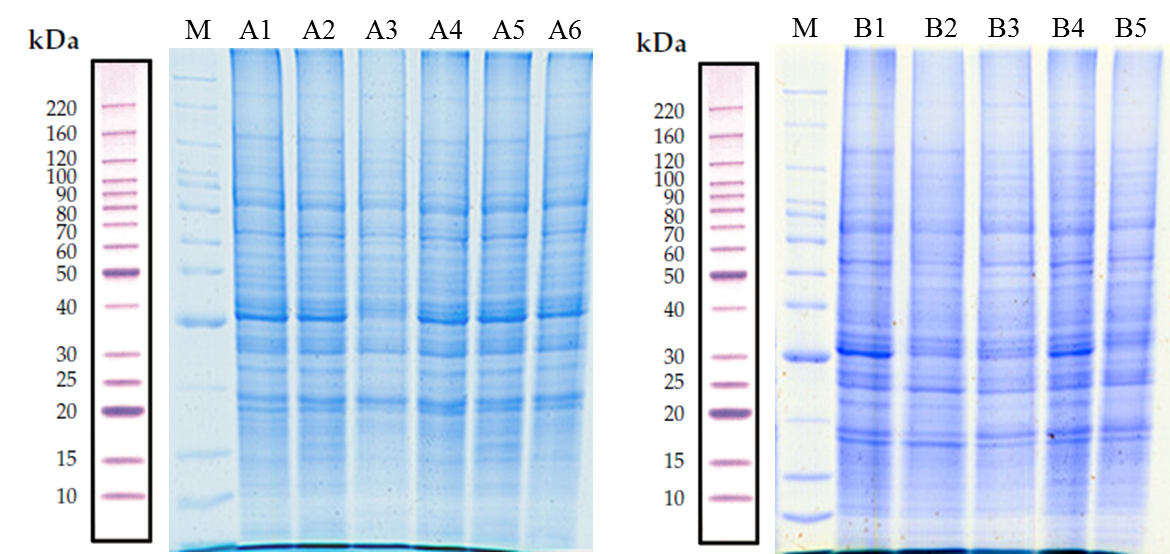


**Supplementary Figure 1.** The SDS-PAGE electrophoretic diagram. A1, GU3Y-1; A2, UG6Y-1; A3, UG9Y-1; A4, UG11Y-2; A5, UG3Y-3; A6, UG6Y-3; B1, UG3Y-2; B2, UG6Y-2; B3, UG9Y-2; B4, UG11Y-3; B5, UG9Y-3.


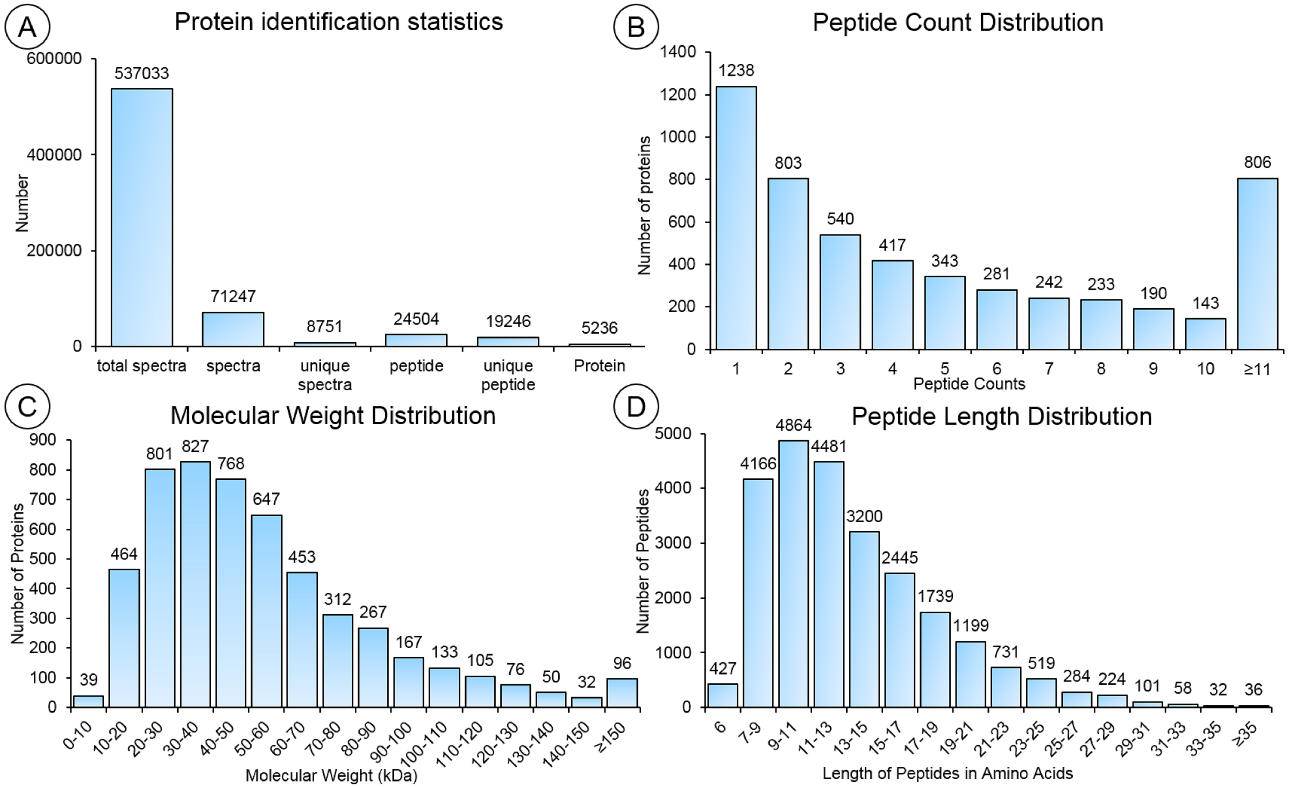


**Supplementary Figure 2.** (A) The identification results of proteins in *E. urograndis*; (B) Number distribution of all identified peptide. The abscissa is the number of peptides, and the ordinate is the number of proteins in this peptide number; (C) Molecular weight (Da) of all identified proteins, (D) Length distribution of all identified proteins.
